# polishCLR: A Nextflow Workflow for Polishing PacBio CLR Genome Assemblies

**DOI:** 10.1093/gbe/evad020

**Published:** 2023-02-16

**Authors:** Jennifer Chang, Amanda R Stahlke, Sivanandan Chudalayandi, Benjamin D Rosen, Anna K Childers, Andrew J Severin

**Affiliations:** USDA, Agricultural Research Service, Jamie Whitten Delta States Research Center, Genomics and Bioinformatics Research Unit, Stoneville, Mississippi; Oak Ridge Institute for Science and Education, Oak Ridge, Tennessee; Genome Informatics Facility, Office of Biotechnology, Iowa State University, Ames; USDA, Agricultural Research Service, Beltsville Agricultural Research Center, Bee Research Laboratory, Beltsville Maryland; Genome Informatics Facility, Office of Biotechnology, Iowa State University, Ames; USDA, Agricultural Research Service, Beltsville Agricultural Research Center, Animal Genomics and Improvement Laboratory, Beltsville, Maryland; USDA, Agricultural Research Service, Beltsville Agricultural Research Center, Bee Research Laboratory, Beltsville Maryland; Genome Informatics Facility, Office of Biotechnology, Iowa State University, Ames

**Keywords:** genome, Nextflow, polish, polishCLR, assembly, QV

## Abstract

Long-read sequencing has revolutionized genome assembly, yielding highly contiguous, chromosome-level contigs. However, assemblies from some third generation long read technologies, such as Pacific Biosciences (PacBio) continuous long reads (CLR), have a high error rate. Such errors can be corrected with short reads through a process called polishing. Although best practices for polishing non-model de novo genome assemblies were recently described by the Vertebrate Genome Project (VGP) Assembly community, there is a need for a publicly available, reproducible workflow that can be easily implemented and run on a conventional high performance computing environment. Here, we describe polishCLR (https://github.com/isugifNF/polishCLR), a reproducible Nextflow workflow that implements best practices for polishing assemblies made from CLR data. PolishCLR can be initiated from several input options that extend best practices to suboptimal cases. It also provides re-entry points throughout several key processes, including identifying duplicate haplotypes in purge_dups, allowing a break for scaffolding if data are available, and throughout multiple rounds of polishing and evaluation with Arrow and FreeBayes. PolishCLR is containerized and publicly available for the greater assembly community as a tool to complete assemblies from existing, error-prone long-read data.

**Significance**

With the advent of cost-efficient DNA sequencing technology, it is now possible to determine the chromosome sequences of a species. Unfortunately, there are still errors in the sequences of these genomes that need correcting. The error correcting best practices have many steps and are susceptible to human error. In this work, we have developed a workflow that makes the error correction process easier to perform and show with an example dataset just how much we can improve the quality of the genome by removing errors through polishing. We named it polishCLR.

Long reads, including those generated by third-generation sequencing platforms such as Pacific Biosciences (PacBio) and Oxford Nanopore Technology (ONT), have revolutionized genome assembly (Childers et al. 2021; Hotaling et al. 2021; Rhie et al. 2021). However, until recent advances (Hon et al. 2020; Wang et al. 2021), long-read sequencing technologies have had high error rates (5–15%), especially among indels (Watson and Warr 2019). Thus, a large proportion of long-read data that is currently publicly available yields assemblies with low overall consensus accuracy, which, if left uncorrected, negatively impacts downstream analyses, such as gene annotation (Watson and Warr 2019). These assembly errors require correction with an additional higher fidelity read set, such as short-read Illumina data, in a process called polishing (Walker et al. 2014; Chin et al. 2016; Hepler et al. 2016).

Polishing can be a complex process, with high computational cost, non-trivial file-handling, and issues around special cases that must be resolved. For example, the long-read contig assembly should ideally be polished with high-fidelity reads from the same individual, but this may not be technically feasible when sufficient DNA cannot be extracted from individual specimens, for example in small-bodied organisms such as many insects. In such cases, it is necessary to modify parameters in a standard workflow. Best practices for de novo, chromosome-scale vertebrate genome assembly from error prone PacBio continuous long reads (CLR) reads were recently described (Rhie et al. 2021), however it can be challenging to run this code and reproduce widely. In order to produce the best possible genome assemblies using existing data from species regardless of their position in the tree of life, the genome assembly community needs a publicly available, flexible and reproducible workflow that is containerized so it can be run on any conventional high performance computing (HPC) environment.

Bioinformatic pipelines with complex entrance and decision points, such as polishing, are inherently difficult to track, develop, and debug. Increasing interest in workflow development systems that track data and software provenance, enable scalability and reproducibility, and re-entrant code (Wratten et al. 2021) have led to the development of several workflow languages, largely inspired by GNU Make (Stallman and McGrath 1991; Köster and Rahmann 2012; Amstutz et al. 2016). Nextflow is a Domain Specific Language (Di Tommaso et al. 2017) that currently leads workflow systems in terms of ease of scripting and submitting to cloud computing resources (Fjukstad and Bongo 2017; Leipzig 2017; Spjuth et al. 2020; Jackson et al. 2021). A key benefit of Nextflow compared with earlier workflow languages is being able to submit jobs to a local machine, an HPC, or cloud-based compute environments. These features empower a large range of bioinformatic pipelines, for example, initial read processing and annotation lift-over (Federico et al. 2019; Talenti and Prendergast 2021). In this paper, we describe polishCLR, a reproducible Nextflow workflow which implements the current best practices for polishing CLR assemblies and is flexible to multiple input assembly and sample considerations.

The polishCLR workflow can be easily initiated from three input cases (fig. 1). Users may start with an unresolved primary assembly without associated contigs by default in Case 1 (e.g., the output of Canu or wtdbg2 Koren et al. 2017; Ruan and Li 2020). Additionally, it can handle a haplotype-resolved but unpolished set (Case 2) (e.g., the output of FALCON-Unzip 3-unzip [Chin et al. 2016]). In the ideal case (Case 3), the pipeline is initiated with a haplotype-resolved, CLR long-read polished set of primary and alternate contigs (e.g., the output of FALCON-Unzip 4-polish) indicated with—falcon-unzip true. In all cases, inclusion of organellar genomes, for example the mitochondrial genome, is required to appropriately polish nuclear mitochondrial or plasmid pseudogenes (Howe et al. 2021). Organellar genomes must be generated and polished separately, using pipelines such as the mitochondrial companion to polishCLR, mitoPolishCLR (Formenti and Stahlke 2022) or mitoVGP (Formenti et al. 2021).

**Fig. 1.**
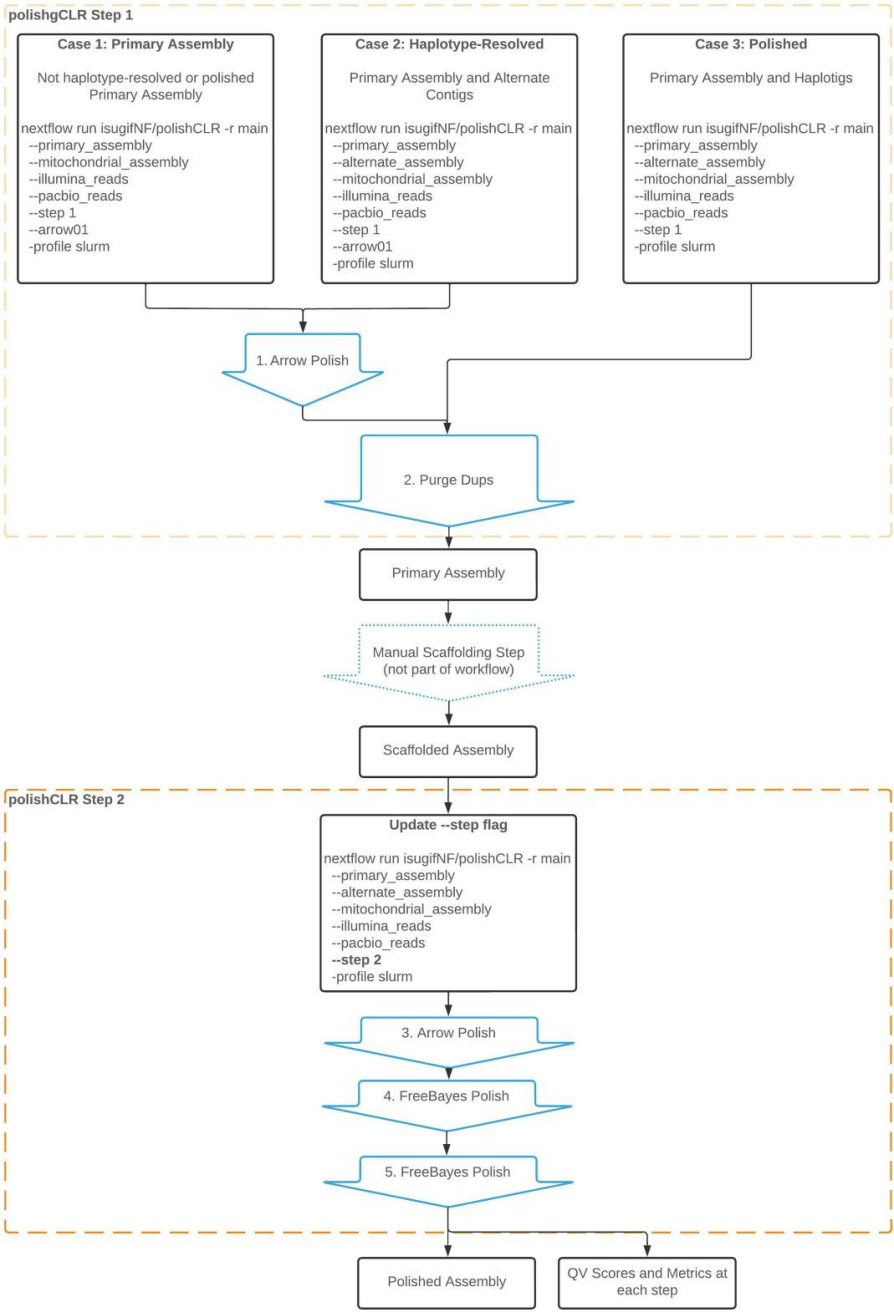
Diagram of polishCLR workflow for three input cases. Polishing Steps 1 and 2 are run separately to accommodate an optional scaffolding step. Labeled arrows indicate processes while solid boxes indicate products. The dotted arrow indicates that the manual scaffolding step is optional and not within the scope of this pipeline.

The workflow is divided into two steps, controlled by a—step parameter flag, which allows the user to scaffold contigs before the second round of polishing and therefore gap-fill across correctly oriented scaffolded contigs.

In Step 1, if initiating the workflow under Case 1 or 2, unpolished primary contigs are merged with the organellar genome and alternate haplotypes if available, then polished with a single round of Arrow long-read polishing (Pacific BioScience) before entering the core workflow. During Arrow steps (here and later in Step 2), polishCLR improves re-entry and computational resource use by delineating at least seven Nextflow processes: 1) indexing each contig, 2) creating a pbmm2 index of the assembly, 3) aligning PacBio reads to the assembly, 4) submitting a GCpp Arrow job for each contig in parallel, 5) combining the separate contig variant calling format (VCF) files, 6) reformatting Arrow generated VCF for Merfin filtering (Formenti et al. 2021, 2022), and 7) converting the resultant VCF back to FASTA format. Then, in all three cases, the core workflow employs purge_dups v. 1.2.5 (Guan et al. 2020) to remove duplicated sequence at the ends of separated primary contigs, with cutoffs automatically estimated from a generated histogram of long-read coverage. The histogram is captured as one of the relevant outputs for users to review. Purged primary sequences are then concatenated to the alternate haplotype contigs and the combined alternate set is purged of duplicates. BUSCO completeness metrics (Simao et al. 2015; Waterhouse et al. 2018; Seppey et al. 2019; Manni et al. 2021) are generated for the primary contigs before and after removing duplicated content to ensure that cutoff parameters are effective and do not remove too much genic content. The eukaryotic BUSCO database is used by default, but users may provide a designated lineage (controlled by a busco-lineage flag). If additional data are available, this de-duplicated primary contig assembly can then be scaffolded by the user before initiating the second phase of the workflow, for example with Hi-C data (Lieberman-Aiden et al. 2009; Durand et al. 2016) and YaHS (Zhou et al. 2023).

In Step 2, the primary, alternate, and organellar assemblies are merged and polished with an additional round of Arrow which can perform gap-filling, followed by two rounds of FreeBayes (Garrison and Marth 2012). Indeed, the iterative nature of polishing benefits from the re-entrant caching and templates of the workflow. By default, this second round of Arrow-identified variants are only filtered via Merfin if the CLR and the Illumina reads came from the same specimen, adding additional Nextflow processes to the Arrow delineation described above to create a meryl genome database and perform filtering (Formenti et al. 2021). However, if short-read data are from a different specimen than the long-read-based contig assembly, then Merfin filtering can be turned off to avoid over-polishing with the parameter flag same-specimen false. As with Arrow, polishCLR takes advantage of Nextflow in seven processes to implement FreeBayes: 1) creating contig windows, 2) generating a meryl database from the genome, 3) aligning Illumina short reads, 4) polishing via FreeBayes, 5) combining windowed VCF files, 6) filtering VCFs by Merfin, 7) and converting VCFs to FASTA. Throughout the polishCLR workflow, de novo evaluation reports are automatically generated to assess genome assembly quality, including k-mer based completeness and consensus accuracy QV scores via Merqury (Rhie et al. 2020), as well as genome size distribution statistics generated with BBMap (e.g., N50) (Bushnell 2014). These reports allow users to understand how the assembly changed through each major phase of the workflow. If a reference is available, reference-based evaluation methods, such as identification of mis-assemblies provided by QUAST-LG (Mikheenko et al. 2018) may also be valuable. The complete, detailed pipeline can be viewed in supplementary figure S1, Supplementary Material online.

The polishCLR workflow is publicly available (https://github.com/isugifNF/polishCLR), reproducible, interoperable, easily portable, and can be run on a conventional HPC or extended to cloud computing resources simply by swapping out the Nextflow config file. Software dependencies are listed in a conda environment file. Its use has been demonstrated on publicly available arthropod species assemblies, such as *Helicoverpa zea* (Stahlke et al. 2022) and *Pectinophora gossypiella* (GenBank accession GCA_024362695.1; Stahlke et al. 2023), generated as part of the Ag100Pest Initiative (Childers et al. 2021). Runtimes and summaries from each of the three starting input cases are included (supplementary table S1, Supplementary Material online; Stahlke and Coates 2022). We also provide test data from both CLR and Illumina reads of Chromosome 30 of *H. zea* (GenBank accession GCA_022581195.1) to test performance (Stahlke and Coates 2022).

Error-correction continues to be an important component of accurate genome assembly, however data from distinct sequencing technology strategies present distinct error-correction needs that require distinct workflows. For example, PacBio High Fidelity (HiFi) circular consensus reads can be improved by correcting homopolymer errors (Wenger et al. 2019; Shafin et al. 2021; McCartney et al. 2022), and polishing with ONT reads benefits from analyzing the raw signal from the sequencer (Loman et al. 2015; Lee et al. 2021). The polishCLR pipeline will increase the efficiency of polishing genomes assembled from CLR reads and reduce the potential of human error in this multistep process. The workflow is available on GitHub and welcomes future contributions.

## Supplementary Material

evad020_Supplementary_Data

## Data Availability

The code described in the article is publicly available on GitHub at the repository https://github.com/isugifNF/polishCLR and documented with ReadTheDocs at https://isugifnf.github.io/polishCLR/, last updated November 8, 2022, and has been archived on Zenodo (https://zenodo.org/record/7306043#.Y2rXT3bMJD8), last updated November 8, 2022. Raw sequence reads for the example dataset of *H. zea* have been deposited to NCBI under PRJNA804956. Example input genome assemblies of *H. zea* have been deposited on the Ag Data Commons, available at https://doi.org/10.15482/USDA.ADC/1524676, last updated November 8, 2022.
