# Supplementary material for: polishCLR: A Nextflow Workflow for Polishing PacBio CLR Genome Assemblies
Source: Genome Biol Evol. 2023 Feb 16;15(3):evad020. doi: 10.1093/gbe/evad020 (PMC9985148; doi:10.1093/gbe/evad020)
Supplement: evad020_Supplementary_Data [file evad020_supplementary_data.docx]

**Supplemental Figures and Tables for "polishCLR: a Nextflow workflow for polishing PacBio CLR genome assemblies"**

**Supplemental Figure 1.** Detailed diagram of the polishCLR pipeline. PacBio CLR long reads, Hi-C data, Illumina short reads, and organellar input data are shown in the green boxes.

**Supplemental Table 1.** The polishCLR workflow was benchmarked on the primary contigs of *Helicoverpa zea* generated by FALCON (Chin et al. 2016).


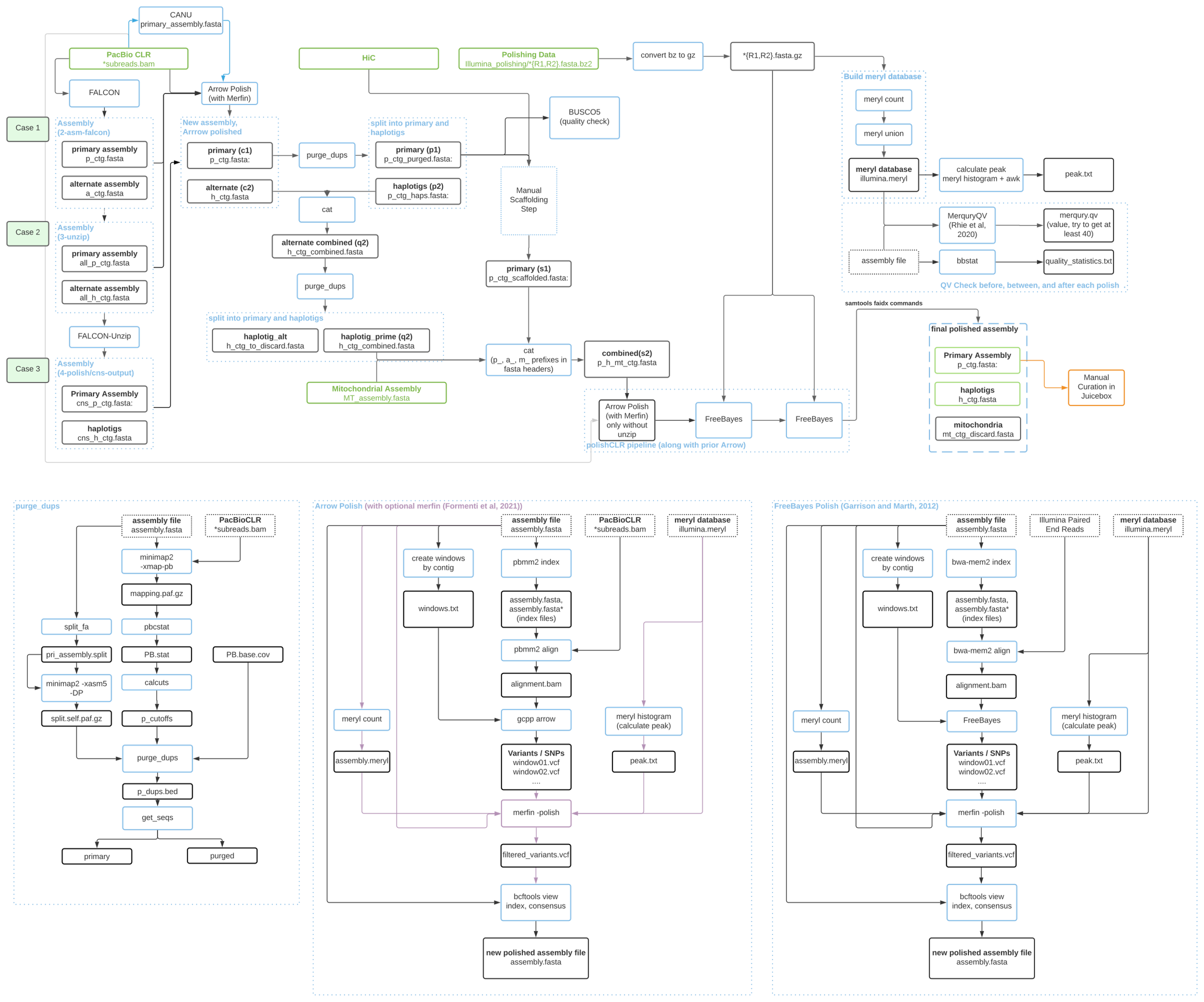


**Supplemental Figure 1.** Detailed diagram of the polishCLR pipeline. PacBio CLR long reads, Hi-C data, Illumina short reads, and organellar input data are shown in the green boxes. A detailed view of the Arrow and FreeBayes polish steps are expanded below separately, with the optional Merfin filtering during the Arrow polishing step shown in purple. Merfin filtering is part of all FreeBayes polishing steps.

| Case | Input stage of Falcon assembly | Input Genome Size (Mb) / Number of contigs | Starting QV | Final QV | CPU hours | Output Genome Size (Mb) / Number of contigs |
| --- | --- | --- | --- | --- | --- | --- |
| 1 | 2-asm-falcon/ | 501.287 / 789 | 31.8218 | 40.3033 | 211.3 | 500.578 / 799 |
| 2 | 3-unzip/ | 515.499 / 1125 | 31.8492 | 38.9997 | 224.2 | 511.878 / 1022 |
| 3 | 4-polish/ | 509.063 / 882 | 38.8556 | 41.9163 | 195.0 | 509.052 / 882 |

**Supplemental Table 1.** The polishCLR workflow was benchmarked on the primary contigs of *Helicoverpa zea* generated by FALCON (Chin et al. 2016). Metrics for each assembly include starting pseudo-haploid primary and alternate combined genome size (Mb) and number of contigs, initial quality scores, CPU hours through the pipeline final quality scores, and final genome size and number of contigs. This table provides an indication of scalability of the pipeline on a SLURM managed HPC.
